# Conceptualization and validation of the TILT questionnaire: relationship with IGD and life satisfaction

**DOI:** 10.3389/fpsyg.2024.1409368

**Published:** 2024-07-08

**Authors:** Iván Bonilla, Andrés Chamarro, Phil Birch, Benjamin T. Sharpe, Adrián Martín-Castellanos, Diego Muriarte, Carles Ventura

**Affiliations:** ^1^Sports Research Institute, Autonomous University of Barcelona, Barcelona, Spain; ^2^Institute of Applied Sciences, University of Chichester, Chichester, United Kingdom; ^3^Institute of Psychology, Business, and Human Sciences, University of Chichester, Chichester, United Kingdom; ^4^Alfonso X El Sabio University, Madrid, Spain; ^5^National Institute of Physical Education of Catalonia – University of Barcelona, Barcelona, Spain

**Keywords:** eSports, psychometrics, emotion, performance, health, internet gaming disorder

## Abstract

Currently, the study of esports is growing within the field of psychology. Among the different variables attracting interest — including stress or psychological factors associated with performance — an emerging concept known as *tilt* is gaining prominence in the literature. However, this construct has yet to be operationalized or defined. Thus, the present study aims to address this gap by defining and conceptualizing TILT while devising and validating a questionnaire to measure the construct in esports players. The initial phase of the study comprised 27 interviews conducted with professional players (*n* = 6), semi-professionals (*n* = 8), amateurs (*n* = 8), and coaches (*n* = 5) to characterize the concept of tilt. Following these interviews, a definition of tilt was formulated, and a panel of five experts in sports psychology and esports proposed a comprehensive set of 53 items. A total of 488 participants (278 males, 210 females), aged 18–50 (mean age = 26.9 years, SD = 7.57), completed the survey, including the 53 tilt items, a questionnaire measuring toxic behavior, and the Internet Gaming Disorder Scale-Short Form (IGDS9-SF). The tilt construct is primarily characterized as a state of frustration escalating into anger, resulting in diminished performance, attention, and recurring negative thoughts about errors. Its onset typically coincides with stressful situations, persisting for approximately 30 min. Through an Exploratory Factor Analysis (EFA), 18 items were retained and categorized into two factors: Causes (7 Items) and Consequences (11 Items) of tilt. The entire questionnaire yielded a Cronbach’s α of 0.922, with the first and second factors showing values of 0.854 and 0.890, respectively. Confirmatory factor analysis (CFA) revealed an acceptable fit for the 2-factor solution. Correlations with related constructs, such as Toxic Behavior and IGD, provided preliminary evidence of external validity. Empirical evidence for the validity and internal consistency of the Tilt Scale is robust, indicating its potential utility in future research on the psychological experiences of esports players.

## Introduction

The realm of esports is experiencing rapid expansion, as projected figures for 2025 anticipate a significant upswing in both regular subscribers (318 million) and casual viewers (322.7 million). This reflects a notable 19.12% increase from the preceding year (Global eSports market size 2023 and Gough, 2024). Concurrently, research in this domain has witnessed consistent growth over the past decade (Reitman et al., 2020), with scholarly investigations spanning diverse areas such as economics (e.g., Cranmer et al., 2021) and sports science (e.g., Sharpe et al., 2022, 2024a,b). This burgeoning body of research has engendered discussions regarding the multifaceted fields of expertise implicated in esports, marking the initial strides toward formalizing its ontology within the realm of scientific inquiry (Brock, 2023).

In the domain of psychology, particularly within the field of sports psychology, esports and its psychological components have garnered significant attention within the scientific community. Numerous investigations have delved into various facets, encompassing the identification of noteworthy stressors (Smith et al., 2019; Leis and Lautenbach, 2020; Poulus et al., 2022a) and their correlation with mental toughness (Poulus et al., 2020). Additionally, research has explored coping strategies (Leis et al., 2022; Poulus et al., 2022b), sleep quality and habits (Klier et al., 2022), the repercussions of winning or losing streaks in competitive scenarios (Machado et al., 2022), as well as their impact on psychophysiological responses (Mendoza et al., 2021) and self-regulation (Trotter et al., 2023). Furthermore, investigations have delved into the psychological factors underpinning sporting performance (Parshakov and Zavertiaeva, 2018; Nagorsky and Wiemeyer, 2020; Sharpe et al., 2022). This includes examining the influence of emotions (Behnke et al., 2022), the requisite psychological skills (Trotter et al., 2021; Bonilla et al., 2022), positive mental health (Griffith and Sharpe, 2024), the role of personality traits (Birch et al., 2023), the impact of high-pressure situations (Sharpe et al., 2024a), and the effects of streaming while gaming on players’ efficiency and in-game behavior over time (Matsui et al., 2020).

The themes currently under investigation in esports exhibit a parallel with subjects extensively studied in sports psychology. Noteworthy examples include the correlation between mental health and performance (Gorczynski et al., 2021), the perspectives of health (Monteiro Pereira et al., 2023), the delineation of crucial psychological skills and their training (Stamatis et al., 2020), skill transfer between esports and traditional sports (Murphy et al., 2020), the use of heart rate variability to index self-regulation (Welsh et al., 2023), and the examination of factors like fundamental needs, attentional control, group cohesion, and decision-making within conventional sporting contexts (Coimbra et al., 2022). However, as the exploration of esports deepens, there is potential for a burgeoning interest in psychological dimensions that either remain understudied or are exclusive to the realm of esports. One such concept, particularly prominent at the professional level, is the phenomenon known as “tilt.” This term is familiar to gamers and esports professionals alike, encapsulating moments of anger and frustration experienced during gameplay and competition. This unique psychological aspect adds a distinctive layer to the understanding of performance dynamics in esports.

The concept of *tilt* is not entirely novel, with its origins tracing back to the era of pinball machines, which featured *tilting* mechanisms designed to detect player movements or attempts to manipulate the game. When such actions were detected, the system would either block the movement of the flippers or penalize the player by reducing scores and bonuses. Additionally, a sign with the word “tilt” is illuminated, signaling to the player to cease such behavior to avoid further consequences (Castle, 2020). While tilt found its initial roots in pinball, it gained widespread usage in poker, particularly with the rise of online poker and its expanding player base and audience. Browne (1989) characterized tilt as a mental state marked by a loss of control, directly influencing a player’s gameplay style, including strategic decisions, gambling, risk-taking, and endurance through prolonged losing streaks. This “tilted” state was associated with significant monetary losses and correlated with various psychological disorders such as depression, anxiety, and sleep disturbances (Palomäki et al., 2013), even potentially exacerbating gambling disorders (Moreau et al., 2020). Moreover, the duration of this mental state could range from minutes to days and, in exceptional cases, persist for months (Browne, 1989). Tilt in poker often elicits negative emotions such as anger or frustration, which are typically inadequately managed, underscoring the pivotal role of emotional regulation in mitigating tilt (Palomäki et al., 2012). This behavior is often associated with other factors such as substance abuse (e.g., alcohol), extended gambling sessions in attempts to recoup losses, or experiencing prolonged losing streaks (Browne, 1989; Palomäki et al., 2013). Certain individual characteristics, such as high emotional sensitivity or diminished perception of defeat, may exacerbate or reduce the intensity of tilt (Palomäki et al., 2013). To further understand and assess the extent of tilt experienced by poker players, Moreau et al. (2017) devised a questionnaire with 21 items, designed to measure the degree of tilt experienced during poker gameplay, dividing the experience of tilt in two main factors: (a) emotional and behavioral tilt, focusing on irritability, anger and sadness and (b) cognitive tilt, focusing on self-control and bet risk-taking.

Despite the notable impact of “tilt” on the performance and psychological well-being of esports players, its exploration from a psychological perspective has been relatively limited. Emerging evidence suggests that esports athletes perceive the avoidance of negative emotions as crucial to their successful performance, a sentiment that aligns with the characteristics of the tilt phenomenon (Poulus et al., 2022b). In a systematic review centered on emotions and emotional regulation within esports, Beres et al. (2023) underscore the significance of acquiring skills to regulate frustration, anger, and tilt. Similarly, Bonilla et al. (2022) emphasize the imperative nature of learning to manage tilt by cultivating emotional control, given its substantial impact on both performance and psychological well-being. The primary triggers for tilt in esports appear to revolve around consecutive losses or errors made by teammates, inducing emotional states characterized by anger, anxiety, and stress. These emotional responses may escalate to a point where players contemplate abandoning the game (Wu et al., 2021; Sharma et al., 2022) or engage in toxic behaviors such as trash-talking, intentional abandonment, or cheating (Türkay et al., 2020). As we have seen, tilt is a construct that generates a great impact on the performance and well-being of players, its central axis being emotions related to anger and frustration. In any case, the behaviors are not clear, giving rise to other behaviors such as toxicity, decision making or stress, as possible related behaviors.

### Study aims

**Figure 1:**
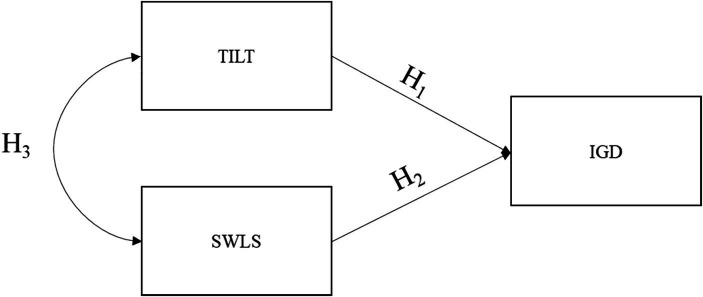
Model proposed for this study. SWLS, life satisfaction; IGD, internet gaming disorder.

The study aims to establish a comprehensive definition of tilt, elucidating its key characteristics and underlying structure to provide a unified framework guiding future research. Secondly, the study endeavors to develop a psychometric instrument capable of effectively measuring tilt. Lastly, the investigation seeks to explore the relationship between tilt and other pertinent constructs, as illustrated in Figure 1, including internet gaming disorder (IGD; Pontes and Griffiths, 2014) and satisfaction with life (SWLS; Diener et al., 1985). Previous research has shown that Internet Gaming Disorder is linked to a heightened prevalence of psychopathology and impulsivity, alongside diminished levels of life satisfaction and self-esteem (Bargeron and Hormes, 2017). Moreover, these impacts are particularly pronounced in the life satisfaction of teenagers and young adults (Phan et al., 2019; Teng et al., 2020). Nevertheless, the exact nature of the relationships between these variables remains unclear, thereby presenting an opportunity to identify behaviors closely associated with gaming that may serve as early indicators of problematic gaming habits. Consequently, the current study not only establishes a connection between Tilt and IGD or life satisfaction for validation purposes, but also considers Tilt as a potential precursor variable to IGD, offering valuable insights for the development of future prevention and intervention strategies.

The study posits several hypotheses. Firstly, it hypothesizes a positive relationship between TILT and IGD (H_1_). Additionally, the study suggests a negative relationship between Life Satisfaction and IGD (H_2_), and finally, it posits a negative relationship between TILT and Life Satisfaction (H_3_).

## Materials and methods

### Participants

All participants in the study were individuals proficient in the Spanish language, encompassing both video game enthusiasts and esports players, as well as coaches within the esports domain. In the first phase, 32 semi-structured interviews were conducted. The participants were selected through convenience sampling from international professional and amateur clubs. The inclusion participants were (a) to have participated in a national or international competition in the last split or 3 months, (b) to be part of a club or esports organization and (c) to be training the last month at least 5 days per week or played a minimum of 15 h of ranked matches (Mendoza et al., 2023). The data collection process stopped when information saturation was detected, because enough data was collected for the conclusions and interviews does not give us new information. Five of the initial interviews were excluded after transcription because they did not provide sufficient information when analyzing the preliminary results, leaving 27 participants (Men = 18, Women = 9) with a mean age of 21.7 years (SD =7.91) and 3.2 years (SD = 1,64) of experience. The sample consisted of professional (*N* = 6), semi-professional (*N* = 8), amateur (*N* = 8), and coach (*N* = 5) players. All data were collected in the third trimester of 2022. In the second phase, a sample calculation using G*Power (version 3.1) software was done, and the minimum needed to make the psychometric analysis and equation model was 223 (Faul et al., 2007; Anthoine et al., 2014). Snowball sampling was employed on discord official clubs and videogames servers, twitter, reedit and mediavida forums, also direct contact with professional and amateur clubs, associations and leagues was made yielding 528 responses, if participants had less than 5 h of playing every week, they were excluded from the study (Mendoza et al., 2023). After debugging the data (i.e., anomalous responses, extreme cases, blank responses, and repeated responses), 488 participants were included in the psychometric study (56.97% men and 43.03% women) with a mean age of 26.9 years (SD = 7.57), dedicating a mean of 3.91 h (SD = 6.82) per day to playing videogames. Participants disclosed their primary gaming preferences, with 62% engaging in esports and 38% playing video games, having a mean of 4.54 years of experience (SD = 2.37) with videogames or esports. Data was collected during the second trimester of 2023. In both phases, inclusion and exclusion criteria for participant selection and classification into esports or videogames were based on guidelines proposed by Mendoza et al. (2023). These criteria were utilized to ascertain participants’ status as gamers or esports players and determine their proficiency levels (i.e., professional, semi-professional, or amateur).

### Instrument

A semi-structured interview was conducted in the first phase, lasting approximately 45 min. The interview covered the following topics: (a) participants’ experiences in esports, (b) common experiences related to tilt, (c) key characteristics of tilt, (d) defining the tilt construct, (e) identifying facilitating and protective factors, and (f) exploring the consequences of episodes characterized by high levels of tilt.

In the second phase, participants completed a questionnaire comprising sociodemographic indicators (e.g., gender, age, experience, hours of play per day) along with the following scales.

#### Tilt questionnaire (TILTQ)

**Figure 2:**
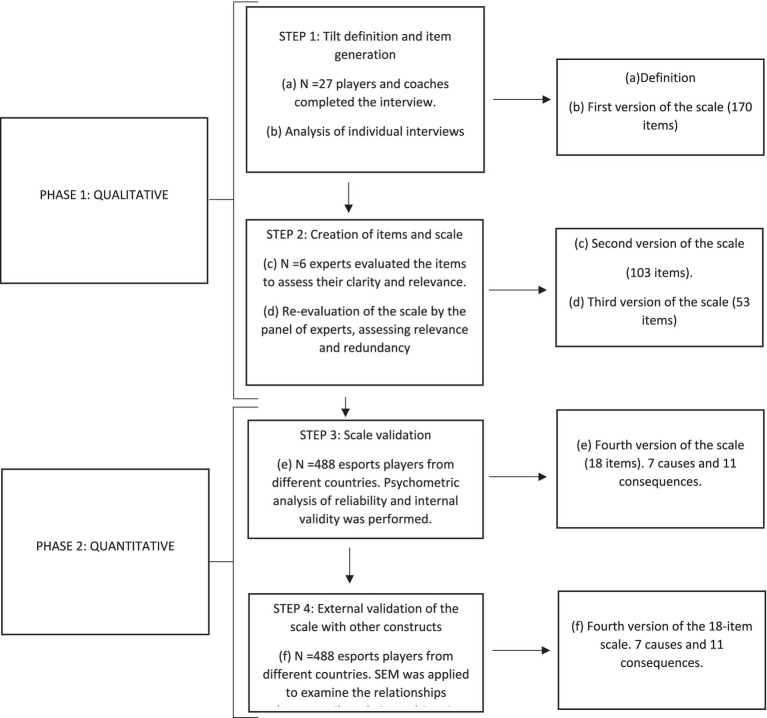
Process of creating the definition of tilt and the measurement scale.

As can be seen in Figure 2, different versions of the questionnaire were constructed during the process of creating the measurement scale. The final version utilized in the study consisted of 18 items (see Table 1), categorized into two dimensions: causes of tilt (comprising 7 items) and consequences of tilt (comprising 11 items; see Table 1 for items), and asked to indicate the extent to which you have experienced the following situations during a game in the last 15 days. Respondents rated each item on a five-point Likert-type scale, ranging from 1 (strongly disagree) to 5 (strongly agree). Total scores ranged from 18 to 90 points, with higher scores indicating greater tilt. In the current investigation, Cronbach’s Alpha coefficients were 0.89 for the causes dimension, 0.89 for the consequences dimension, and 0.92 for the overall tilt scale.

**Table 1:** Items and structure of the TILTQ.

Structure	Factor loadings
**TILTQ** Please indicate the extent to which you have experienced the following situations during a game in the last 15 days.	
**Causes**	
I have lost because of things in the game I could not control. I have failed to make important moves. I have made mistakes in things I know I can do well. I have made wrong decisions. I failed even though I knew what I had to do. I have felt that I have more ability than I have been able to demonstrate. I have played frustrating games.	0.667 0.671 0.695 0.793 0.713 0.799 0.728
**Consequences**	
I have felt that the game was not fair. I have exploded with rage. I have felt irritated. I have made decisions without thinking. I have found it hard to concentrate. I have had mood swings due to the outcome of my games. I have felt that I have no energy. I have felt that I have been on a losing streak that I could not get out of. I have played hastily. I have continued to play even though I did not feel like it. I have written off games as lost.	0.585 0.620 0.758 0.785 0.714 0.764 0.567 0.751 0.731 0.618 0.668

#### Internet gaming disorder (IGD)

IGD was evaluated using the Spanish version of the Internet Gaming Disorder Scale-Short Form (IGDS9-SF; Beranuy et al., 2020). This scale comprises nine items designed to assess the severity of IGD and its impact on online and offline gaming activities over a 12-month period. Each item is rated on a 5-point Likert scale, ranging from 1 (Never) to 5 (Very often). Total scores on the scale can range from 9 to 45, with higher scores indicating a greater risk of IGD. In the present study, the Cronbach’s Alpha coefficient for the IGDS9-SF was 0.83.

*Satisfaction with Life Scale (SWLS).* This self-report questionnaire (Diener et al., 1985) is used to measure overall life satisfaction. Each item is scored on a five-point Likert scale, ranging from 1 (Strongly disagree) to 7 (Strongly agree). Total scores can range from 5 to 35, with higher scores indicating greater life satisfaction. The Cronbach’s Alpha obtained in the present study was 0.81.

### Study design and procedure

A two-phase study was conducted using a mixed-methods design, since, as mentioned above, the variables and factors underpinning tilt have not yet been adequately defined and studied within the field of esports. A qualitative methodology was used (Phase 1), conducting individual interviews with players and coaches — professional, semi-professional, and amateur — in order to establish a definition of the construct and develop a scale to measure tilt. A quantitative methodology was adopted (Phase 2) to carry out the relevant psychometric analysis, providing external validation of the scale with IGD and SWLS to test the various hypotheses (see Figure 2).

The study employed a mixed-methods research design comprising two distinct phases, as delineated in Figure 2. During the first phase, interviews were conducted in the third trimester of 2022. Participants were selected through convenience sampling and were provided with a comprehensive briefing on the study’s aims and procedures, subsequently giving informed consent by signing a consent form. Interviews were administered through both face-to-face interactions and online sessions utilizing platforms such as Discord or Teams. All interview sessions were recorded and subsequently transcribed for the purpose of thematic analysis. Following the interview phase, a precise definition of “Tilt” was formulated, and items for the initial questionnaire were generated. This questionnaire, along with the definition, underwent rigorous evaluation by a panel consisting of six experts (Mage = 42.1; SD = 12.5) in sports psychology, sports science, or esports, with more than 5 years of experience in the field as researchers and practitioners. From an initial pool of 170 items, the expert panel selected 53 items for further consideration.

Moving on to the second phase, an online survey was disseminated via Kobotoolbox during the second trimester of 2023, reaching participants through various channels and social media platforms such as Twitter and Reddit. The survey encompassed gamers of diverse proficiency levels and nationalities, all of whom were Spanish-speaking and capable of responding through mobile devices, tablets, or computers. Prior to initiating the questionnaire, participants were required to review and confirm their agreement with the informed consent statement. In cases of non-consent, participants were courteously directed to the survey closure page and thanked for their time. All data collected were securely stored in an anonymous and encrypted format within the university database of the principal investigator (PI). Access to any identifying information was strictly restricted to the PI alone, ensuring confidentiality and data security in strict adherence to the guidelines set forth by the American Psychological Association (2020). Moreover, ethical approval for the study was obtained from the Research Ethics Committee and awarded by the lead institution (CEEAH 5525).

### Data analysis

In the first phase, a thematic analysis was conducted to categorize the various responses obtained, utilizing the ATLAS.ti software. Following the classification of themes, a series of definitions and key concepts were formulated, serving as the basis for creating the questionnaire items. Subsequently, the same panel of experts described before individually assessed the definitions and items pertaining to the tilt concept. During the item selection process following the guidelines proposed by Lynn (1986), items receiving unanimous agreement from all six experts proceeded directly to the next phase. In contrast, those with between 3 and 5 agreements underwent further review, incorporating suggestions provided by the experts, and making a second round where if 5 experts agreed the item has been included. Finally, items receiving fewer than three affirmative responses were eliminated. Additionally, suggestions for new items were allowed to enhance the item pool. This iterative procedure continued until the final version comprising 53 items was obtained and subjected to psychometric analysis.

In the second phase, the psychometric properties of the TILTQ instrument were assessed. Item-total analysis was carried out, while skewness and kurtosis were calculated to check the normality of the data. Subsequently, an exploratory factor analysis (EFA) with Oblimin rotation was conducted, as suggested by Lloret-Segura et al. (2014), to determine the factor structure. Items with factor loadings below 0.4 or loading on another dimension were eliminated. Additionally, a scree plot was utilized to determine the number of dimensions.

Once the factors and their component items had been selected, confirmatory factor analysis (CFA) was conducted using conventional fit indices, including Comparative Fit Index (CFI) > 0.9, Tucker-Lewis Index (TLI) > 0.9, Root mean square error of approximation (RMESEA) < 0.08, and Goodness of Fit Index (GFI) > 0.9 (Browne and Cudeck, 1993; Marsh et al., 2005). A correlation matrix between IGD, tilt, and SWLS was generated to assess external validity. Finally, structural equation modeling was employed to test the proposed hypotheses, adhering to the same fit criteria as those adopted for the CFA.

All analyses were conducted using JASP 0.18.1.0 statistical software (JASP Team, 2023).

## Results

The results of the exploratory thematic analysis, summarizing the concepts and themes associated with tilt, are presented in Table 2. Two primary dimensions emerged: the causes that trigger tilt and the subsequent consequences experienced once in a tilted state. Participants highlighted that these dimensions fed into each other during the different level states of tilt.

**Table 2:** Main tilt-related themes.

Concept/theme	When it occurs	Quotations
Frustration	When failing, feeling defeated, or when goals are not achieved	“When you are tilted, you feel like nothing is worthwhile, and no matter how much you do, you are not going to achieve your goals.”
Anger	When making mistakes, when teammates do not respond well, and when losing regardless of the amount of time spent playing.	“It is like a snowball that keeps getting bigger and bigger until you finally explode.”
Loss of control	When it is not known why a player wins or loses; it feels like the game is rigged; or experiencing the feeling of playing well but losing anyway.	“The game is often unfair, there are champions who are overpowered, or it is simply impossible to win.”
Decision-making	Situations with multiple failures, tunnel vision, high pressure, and intense competition.	“I have been “tilted” many times when competing, and all of a sudden, I make a move or play in a way that does not make sense.”
Mood swings	In prolonged situations of frustration, anger, and defeats.	“When I start to play, I always feel motivated, but as you tilt, you gradually lose that motivation and end up losing the enthusiasm you had when you began.”
In-game behavior	When faced with repeated failures, the bad behavior of other colleagues or toxic situations.	“When you get tilted, you start doing things you should not, even to the point of being toxic, changing your strategies, or playing just for the sake of it” or “If you are tilted, often you do not stop playing matches because you know that if you win one, the tilt will disappear, but of course when you play tilted you play worse, and you have more chances to keep losing and losing.”

Based on these themes and their components, a definition was formulated and approved by the expert judges. This definition offers a conceptualization of tilt as follows: “Behavior that increases gradually with repeated errors, by oneself or others in a context where performance is required, which generates frustration. This causes anger, emotional lability, decreased performance, attention, and recurrent negative thoughts about the error or defeat. Tilt is closely related to stressful situations, varying from seconds to hours, with an average duration of 30 min.”

An item analysis was conducted before carrying out the exploratory factor analysis of the tilt scale. All items followed a normal distribution, with no outlier responses and no floor or ceiling effects detected. Consequently, all 53 items were retained for further analysis. A comparison of item scores between the upper and lower 25% of the sample revealed significant differences for all items, indicating that the items effectively discriminated between individuals with varying levels of tilt. Before conducting the exploratory factor analysis, the Kaiser-Meyer-Olkin (KMO) index was calculated, yielding a value exceeding 0.9 according to Hutcheson and Sofroniou (1999), this value can be classified as superb. Additionally, Bartlett’s test of sphericity was significant (*X*^2^ = 3706.65; df = 118; *p* < 0.001), confirming the suitability of the data and items for factor analysis.

**Figure 3:**
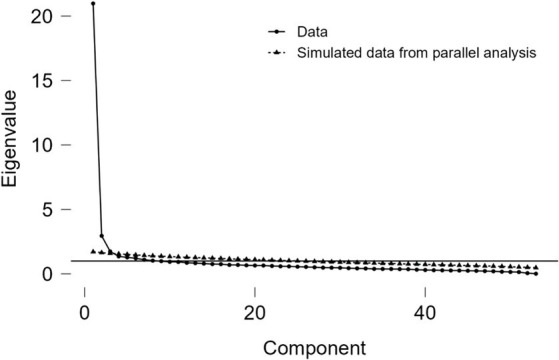
Scree plot showing the initial solution.

An Oblimin rotation was employed for the exploratory factor analysis, anticipating relationships between the potential factors. The scree plot suggested the presence of three factors (see Figure 3).

Upon observing that 10 items had factor loadings below 0.4, they were excluded from the analysis. When evaluating the nine items grouped in the third factor, it was noted that they represented an amalgamation of poorly related concepts and were eliminated. Following these modifications, 34 items were retained for a two-factor solution (eigenvalue >1). However, this solution revealed that 2 items loaded inversely, 8 items loaded on both factors and 6 items loaded below 0.4, resulting in their elimination. Consequently, 18 items remained, with 7 items in the causes factor and 11 in the consequences factor, explaining 51.2% of the variance.

Once the factor structure was determined, reliability was assessed using Cronbach’s Alpha (*α*) and McDonald’s Omega (*ω*) coefficients. For the total tilt scale, McDonald’s Omega was calculated as *ω* = 0.922 (0.912–0.932), while Cronbach’s Alpha was *α* = 0.921 (0.910–0.931). Similarly, for the subscale measuring causes, McDonald’s Omega was *ω* = 0.855 (0.836–0.875), and Cronbach’s Alpha was *α* = 0.854 (0.834–0.873). For the subscale measuring consequences, McDonald’s Omega was *ω* = 0.891 (0.877–0.906), and Cronbach’s Alpha was *α* = 0.890 (0.875–0.904). Based on these results, we can conclude that the total scale and its subscales show adequate reliability indices with scores above 0.70 and less than 0.95, with both subscales scoring less than 0.90 showing not redundancy with a good consistence (Tavakol and Dennick, 2011; Viladrich et al., 2017). The correlation matrix between the total scale and its subscales (see Table 3) shows a high positive correlation.

**Table 3:** Correlation between factors and scale.

Variable	1		2		3
1. TILT causes	–				
2. TILT consequences	0.688	***	–		
3. Total TILT	0.884	***	0.948	***	–

*p < 0.05, ** *p* < 0.01, *** *p* < 0.001.

To assess construct validity, a confirmatory factor analysis (CFA) was conducted using both factors (see Table 1) covariance between factor was 0.81, showing the existence of a general factor called tilt. The model demonstrated acceptable fit indices (*X*^2^ = 484.794; *p* < 0.001), as shown in Table 4, and all factor loadings exceed 0.55 which can be considered good or above (Comrey and Lee, 1992). Given that Byrne (2010) states that the use of both fit indices and factor loadings should be used when assessing factorial validity our results suggest that the proposed model adequately explains the underlying structure of the tilt construct.

**Table 4:** Fit indices of the confirmatory factor analysis.

Index	Value
Comparative Fit Index (CFI)	0.952
Tucker-Lewis Index (TLI)	0.945
Root mean square error of approximation (RMSEA)	0.073

To evaluate convergent validity (see Table 5), it can be observed that the correlations between the tilt scale and its subscales are considerably higher than those observed with other constructs. This indicates that the tilt scale effectively discriminates from related constructs, particularly Internet Gaming Disorder, which could be a regarded as a similar construct since it addresses negative states and consequences related to video gaming. Second, all correlations are statistically significant. Specifically, there is a positive correlation between tilt and IGD and a negative correlation between tilt and life satisfaction. These findings are consistent with theoretical predictions, indicating that the tilt construct behaves as expected in relation to previously established constructs.

**Table 5:** Correlation matrix for the scale and related variables.

Variable	1		2		3		4		5
1. Causes	–								
2. Consequences	0.688	***	-						
3. TILT	0.884	***	0.948	***	-				
4. IGD	0.213	***	0.409	***	0.357	***	-		
5. Satisfaction	−0.339	***	−0.261	***	−0.318	***	−0.315	***	-

*p < 0.05, ** *p* < 0.01, *** *p* < 0.001.

**Figure 4:**
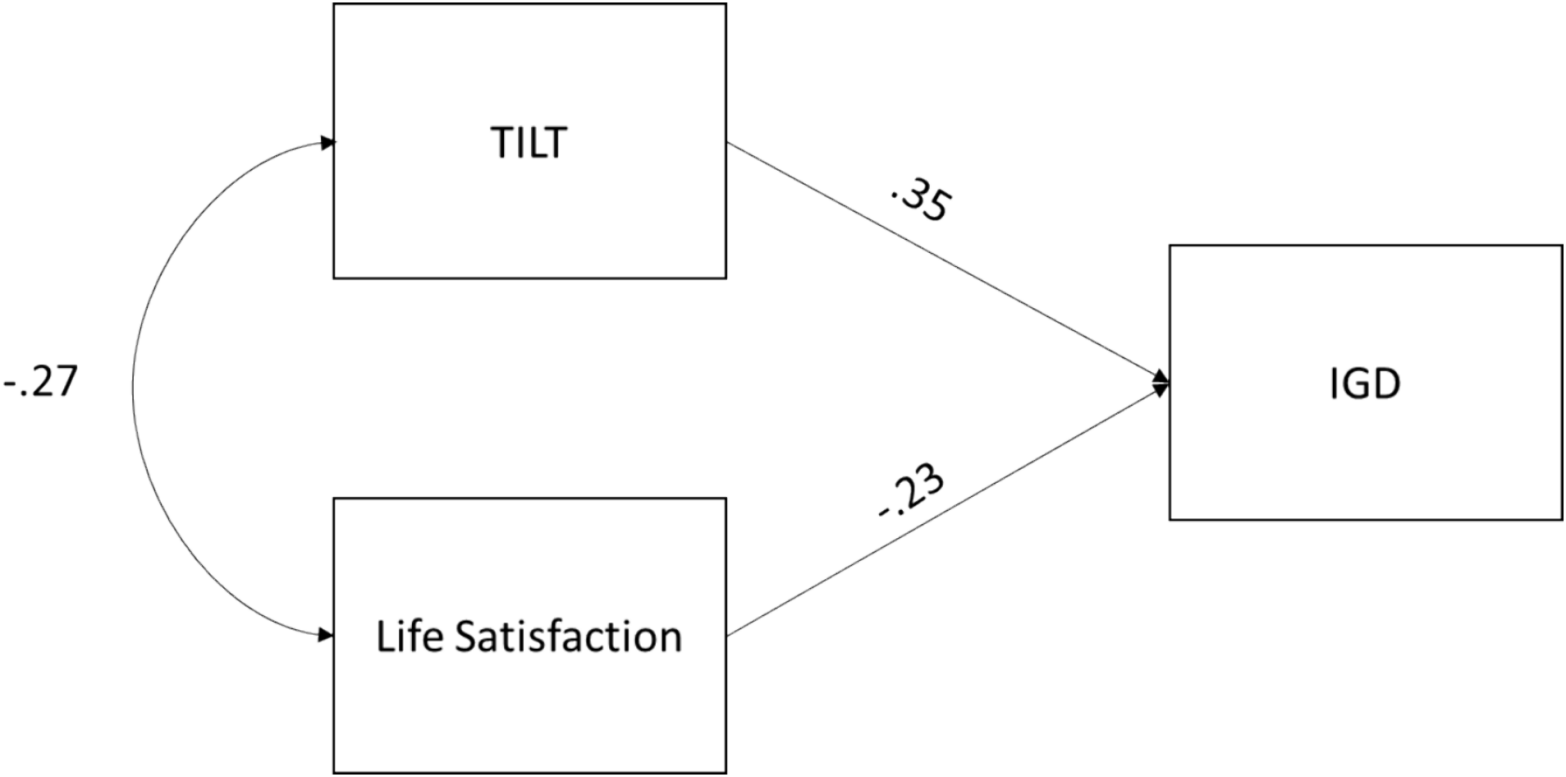
Structural equation model of the variables studied.

Finally, we tested the hypothesized structural equation model for the relationships between tilt, Internet Gaming Disorder, and life satisfaction (see Figure 1). The results indicate an acceptable fit for the model (*X*^2^ = 39.456; *p* < 0.001), providing further evidence of external validity. The model reveals a positive relationship between tilt and IGD, as well as a negative relationship between life satisfaction and IGD. Additionally, a negative covariance between tilt and life satisfaction is evident. The model explains 21% of the variance in IGD (see Figure 4).

## Discussion

The primary aim of this study was to elucidate the concept of tilt, introduce a measurement instrument for the construct, and investigate its association with Internet Gaming Disorder and Life Satisfaction. The initial findings of this research pertain to the proposed definition and components of tilt, as detailed in Table 2. These results suggest that tilt is not an impulsive behavior with an undetermined origin; rather, it exhibits identifiable causes intricately connected to the act of playing video games or participating in esports, particularly within performance-driven scenarios that necessitate the execution of skills to surmount challenges presented by the game. The study revealed that individuals, when faced with the inability to achieve performance goals, undergo a growing sense of frustration that intensifies with prolonged play and repeated attempts to meet their objectives, ultimately triggering the onset of tilt. It is crucial to recognize that the phenomenon of tilt unfolds gradually, “snowballing” over time, often culminating in either explosive manifestations, such as outbursts of anger, or passive expressions, such as a loss of energy and motivation. Adding to the intricacy of tilt is the inclination for individuals experiencing it to persist in gameplay, driven by the hope that achieving victory may alleviate their tilt. Conversely, there is a proclivity for tilted individuals to resort to toxic behaviors, such as quitting the game or engaging in verbal abuse, thereby posing risks to both themselves and others. This complexity in the progression of tilt aligns with prior research in domains like poker (Browne, 1989; Moreau et al., 2017), which shares certain similarities with tilt observed in video games and esports due to the shared underlying logic of gameplay. The study’s findings also resonate with existing research in esports; for instance, Sharma et al. (2022) and Wu et al. (2021) have previously reported tilt-related consequences similar to those identified in the present study, including the inclination to quit games prompted by anger and frustration. Moreover, the research by Türkay et al. (2020) implies that individuals experiencing tilt-like situations are more predisposed to engaging in toxic behaviors or repeated mistakes in performance situations.

Regarding the second aim, the results generated a final 18-item questionnaire, divided into two scales, 7 items for causes and 11 items for consequences (see Table 1 and Suppplementary file).

The questionnaire demonstrates adequate reliability, strong factorial validity with acceptable fit indices, and an explained variance of 51.7%. Additionally, when evaluating external validity, the construct satisfactorily discriminates from other constructs and shows expected relationships IGD and life satisfaction. Consequently, this questionnaire serves as an initially reliable and valid measure for assessing tilt among video game and esports players.

Finally, three hypotheses were formulated to evaluate whether the observed relationships aligned with our expectations, that is, with IGD and life satisfaction to clarify whether tilt and satisfaction are potential predictors of IGD. As depicted in Figure 4, these hypotheses were confirmed, yielding a model that explains 21.7% of the variance. Upon closer examination, it is evident that IGD shows a negative association with life satisfaction, in line with previous research (e.g., Bargeron and Hormes, 2017), and a positive correlation with tilt. Thus, based on the preliminary results, those players prone to high levels of tilt could present a greater risk of developing problematic relationships with video games, which could lead to IGD. Additionally, tilt is found to co-vary with life satisfaction, indicating that esports players experiencing tilt tend to report lower levels of life satisfaction and vice versa. These findings open a new path to understanding the precursor variables involved in Internet gaming disorder, not just the contextual ones or the direct effects on self-esteem, impulsivity or self-esteem (Bargeron and Hormes, 2017), bringing us closer to unraveling the different behaviors that gamers follow to develop a bad relationship with video games or even psychopathology.

These findings pave the way for a new field of study in esports research and opens future lines of research. First, our measurement instrument offers the opportunity to explore the concept of tilt and analyze its relationship with other psychological variables in the context of esports, such as emotional regulation, particularly given that tilt and emotional lability are closely related (Poulus et al., 2022b; Beres et al., 2023), also it allows us to explore its relationship with other cognitive variables like attention or memory (Pedraza-Ramirez et al., 2020). Second, it would be interesting to investigate the relationship between tilt and potentially related variables such as toxicity (Türkay et al., 2020) or the structural characteristics of video games (Wood et al., 2004; Feliu et al., 2023), so we can go further in the understanding of internet gaming disorder specific behaviors. Moreover, it would be useful to develop psychological techniques to mitigate tilt. Such interventions are particularly important to practitioners if we consider the substantial impact of tilt on players and the esports ecosystem; therefore, implementing strategies to reduce individual discomfort, enhance performance, and diminish toxicity could prove highly beneficial to support the overall sustainability of video gaming and esports.

The present study has several limitations that warrant consideration. First, the study sample is limited to a Spanish-speaking culture, which restricts the generalizability of the findings to other cultural contexts. Second, while the tilt instrument effectively measures individual player dimensions, it does not fully capture how teammate behaviors may contribute to tilt. Future versions of the TILTQ could address this limitation by incorporating items specifically designed to assess teammate-induced tilt, thus creating separate versions for individual and team games/esports.

## Conclusion

The present study aimed to bridge the existing gap in research by providing a comprehensive definition and conceptual framework for TILT. In doing so, the study developed and validated a questionnaire designed to effectively measure the construct specifically in esports players. The obtained findings facilitated the conceptualization and quantification of the tilt phenomenon, laying the foundation for exploring its intricate relationships with other variables of interest. With the established validity and internal consistency of the Tilt Scale, this study introduces a valuable tool that holds promise for future research endeavors on the psychological experiences of esports players, transcending diverse cultural contexts. Furthermore, the study paves the way for a novel avenue of research, contributing to an enhanced understanding of this specific behavior within the realms of video gaming and esports.

## Data availability statement

The raw data supporting the conclusions of this article will be made available by the authors, without undue reservation.

## Ethics statement

The studies involving humans were approved by the Research Ethics Committee of the Autonomous University of Barcelona with code CEEAH 5525. The studies were conducted in accordance with the local legislation and institutional requirements. The participants provided their written informed consent to participate in this study.

## Author contributions

IB: Conceptualization, Formal analysis, Methodology, Writing – original draft, Writing – review & editing, Data curation, Funding acquisition, Investigation, Project administration, Software, Supervision, Validation. AC: Conceptualization, Formal analysis, Investigation, Methodology, Supervision, Validation, Writing – original draft, Writing – review & editing. PB: Methodology, Supervision, Validation, Writing – original draft, Writing – review & editing. BS: Methodology, Supervision, Validation, Writing – original draft, Writing – review & editing. AM-C: Formal analysis, Funding acquisition, Supervision, Validation, Writing – original draft, Writing – review & editing. DM: Funding acquisition, Methodology, Supervision, Validation, Writing – original draft, Writing – review & editing. CV: Conceptualization, Formal analysis, Investigation, Methodology, Supervision, Validation, Writing – original draft, Writing – review & editing.

## References

[ref1] American Psychological Association (2020). Publication manual of the American Psychological Association 2020: The official guide to APA style. 7th Edn: American Psychological Association.

[ref2] Anthoine E. Moret L. Regnault A. Sébille V. Hardouin J. (2014). Sample size used to validate a scale: a review of publications on newly-developed patient reported outcomes measures. Health Qual. Life Outcomes 12:176. doi: 10.1186/s12955-014-0176-2, PMID: 25492701 PMC4275948

[ref3] Bargeron A. H. Hormes J. M. (2017). Psychosocial correlates of internet gaming disorder: psychopathology, life satisfaction, and impulsivity. Comput. Hum. Behav. 68, 388–394. doi: 10.1016/j.chb.2016.11.029

[ref4] Behnke M. Gross J. J. Kaczmarek L. D. (2022). The role of emotions in esports performance. Emotion 22, 1059–1070. doi: 10.1037/emo0000903, PMID: 33119343

[ref9003] Beranuy M. Machimbarrena J. M. Asunción Vega-Osés M. Carbonell X. Griffiths M. D. Pontes H. M. (2020). Spanish validation of the Internet Gaming Disorder Scale-Short Form (IGDS9-SF): Prevalence and relationship with online gambling and quality of life. Int. J. Environ. Res. Public Health. 17, 1562–1577. doi: 10.3390/ijerph17051562 32121280 PMC7084394

[ref9002] Beres N. A. Klarkowski M. Mandryk R. L. (2023). Playing with Emotions: A Systematic Review Examining Emotions and Emotion Regulation in Esports Performance. Proceedings of the ACM on Human-computer Interaction, 7, 558–587. doi: 10.1145/3611041

[ref5] Birch P. D. Greenlees L. Sharpe B. T. (2023). An exploratory investigation of personality in counter-strike: global offensive. J. Elect. Gaming Esports 1, 1–9. doi: 10.1123/jege.2022-0027

[ref6] Bonilla I. Chamarro A. Ventura C. (2022). Psychological skills in esports: qualitative study of individual and team players. Aloma 40, 35–41. doi: 10.51698/aloma.2022.40.1.36-41

[ref7] Brock T. (2023). Ontology and interdisciplinary research in esports. Sport, Ethics Philos. 1-17, 1–17. doi: 10.1080/17511321.2023.2260567

[ref8] Browne B. R. (1989). Going on tilt: frequent poker players and control. J. Gambl. Behav. 5, 3–21. doi: 10.1007/bf01022134

[ref9] Browne M. W. Cudeck R. (1993). “Alternative ways of assessing model fit” in Testing structural equation models. eds. Bollen K. A. Long J. S., vol. 21 (Newbury Park, CA: Sage), 230–258.

[ref10] Byrne B. M. (2010). Structural equation modelling with AMOS: Basic concepts, applications, and programming. 2nd Edn. New York: Routledge.

[ref11] Castle P. (2020). Why do pinball machines tilt? Is it allowed? Available at: https://pinballcastle.com/why-pinball-machines-tilt

[ref9004] Cranmer E. E. Han D. D. Van Gisbergen M. Jung T. (2021). Esports matrix: Structuring the esports research agenda. Comput. Human Behav. 117:106671. doi: 10.1016/j.chb.2020.106671

[ref12] Coimbra D. R. Dominski F. H. Brandt R. Bevilacqua G. G. Andreato L. V. Andrade A. (2022). Twenty years of scientific production in sport and exercise psychology journals: a bibliometric analysis in web of science. J. Sport Psychol. 31, 245–261.

[ref13] Comrey A. L. Lee H. B. (1992). A first course in factor analysis. 2nd Edn. Mahwah, NJ: Lawrence Erlbaum Associates.

[ref14] Diener E. Emmons R. A. Larsen R. J. Griffin S. (1985). The satisfaction with life scale. J. Pers. Assess. 49, 71–75. doi: 10.1207/s15327752jpa4901_13 16367493

[ref15] Faul F. Erdfelder E. Lang A. Buchner A. (2007). G*power 3: a flexible statistical power analysis program for the social, behavioral, and biomedical sciences. Behav. Res. Methods 39, 175–191. doi: 10.3758/bf03193146, PMID: 17695343

[ref16] Feliu A. C. Carbonell X. Fúster H. (2023). Diseño de una taxonomía para el estudio psicológico de las características estructurales de los videojuegos. Aloma 41, 37–48. doi: 10.51698/aloma.2023.41.2.37-48

[ref9005] Global eSports market size 2023 Gough C. (2024). Statista. https://www.statista.com/statistics/1256162/global-esports-market-size/

[ref17] Gorczynski P. Currie A. Gibson K. Gouttebarge V. Hainline B. Castaldelli-Maia J. M.. (2021). Developing mental health literacy and cultural competence in elite sport. J. Appl. Sport Psychol. 33, 387–401. doi: 10.1080/10413200.2020.1720045

[ref18] Griffith O. J. Sharpe B. T. (2024). Investigating psychological disparities across gamers: a genre-based study. J. Elect. Gaming Esports 2, 1–9. doi: 10.1123/jege.2023-0040

[ref19] Hutcheson G. Sofroniou N. (1999). The multivariate social scientist. London: Sage Publications Ltd.

[ref20] JASP Team. JASP (Version 0.18.1.0) [Computer software]. (2023). Available at: https://jasp-stats.org/download/

[ref21] Klier K. Seiler K. Wagner M. (2022). Influence of esports on sleep and stress. Z. Sportpsychol. 29, 95–103. doi: 10.1026/1612-5010/a000368

[ref22] Leis O. Lautenbach F. (2020). Psychological and physiological stress in non-competitive and competitive esports settings: a systematic review. Psychol. Sport Exerc. 51:101738. doi: 10.1016/j.psychsport.2020.101738

[ref23] Leis O. Lautenbach F. Birch P. D. Elbe A. M. (2022). Stressors, associated responses, and coping strategies in professional esports players: a qualitative study. Int. J. Esports 3, 1–11. doi: 10.1123/jege.2023-0002

[ref24] Lloret-Segura S. Ferreres-Traver A. Hernández-Baeza A. Tomás-Marco I. (2014). El Análisis Factorial Exploratorio de los Ítems: una guía práctica, revisada y actualizada. Ann. Psychol. 30, 1151–1169. doi: 10.6018/analesps.30.3.199361

[ref25] Lynn M. R. (1986). Determination and quantification of content validity. Nurs. Res. 35:382???386. doi: 10.1097/00006199-198611000-00017 3640358

[ref26] Machado S. de Oliveira Sant'Ana L. Cid L. Teixeira D. Rodrigues F. Travassos B.. (2022). Impact of victory and defeat on the perceived stress and autonomic regulation of professional eSports athletes. Front. Psychol. 13:987149. doi: 10.3389/fpsyg.2022.987149, PMID: 36092047 PMC9454608

[ref27] Marsh H. W. Hau K. T. Grayson D. (2005). “Goodness of fit in structural equation models” in Contemporary psychometrics: A festschrift for Roderick P. McDonald. eds. Maydeu-Olivares A. McArdle J. J. (Lawrence Erlbaum Associates Publishers), 275–340.

[ref28] Matsui A. Sapienza A. Ferrara E. (2020). Does streaming esports affect players' behavior and performance? Games Culture 15, 9–31. doi: 10.1177/1555412019838095

[ref30] Mendoza G. Bonilla I. Chamarro A. Jiménez M. (2023). The defining characteristics of esports players. A systematic review of the samples used in esports research. Aloma 41, 111–120. doi: 10.51698/aloma.2023.41.1.111-120

[ref31] Mendoza G. Clemente-Suárez V. J. Alvero-Cruz J. R. Rivilla I. García-Romero J. Fernández-Navas M.. (2021). The role of experience, perceived match importance, and anxiety on cortisol response in an official esports competition. Int. J. Environ. Res. Public Health 18:2893. doi: 10.3390/ijerph18062893, PMID: 33808997 PMC8000917

[ref32] Monteiro Pereira A. Bolling C. Birch P. D. J. Figueiredo P. Verhagen E. Brito J. (2023). Perspectives of elite esports players and staff members regarding the effects of esports on health – a qualitative study. Sports Medicine–Open 9, 1–17. doi: 10.21203/rs.3.rs-2784247/v1 37493766 PMC10371963

[ref33] Moreau A. Chauchard É. Sévigny S. Giroux I. (2020). Tilt in online poker: loss of control and gambling disorder. Int. J. Environ. Res. Public Health 17:5013. doi: 10.3390/ijerph17145013, PMID: 32668576 PMC7400001

[ref34] Moreau A. Delieuvin J. Chabrol H. Chauchard E. (2017). Online poker tilt scale (OPTS): creation and validation of a tilt assessment in a French population. Int. Gambl. Stud. 17, 205–218. doi: 10.1080/14459795.2017.1321680

[ref35] Murphy C. P. Wakefield A. Birch P. D. J. North J. S. (2020). Esport expertise benefits perceptual-cognitive skill in (traditional) sport. J. Expertise 3, 227–237.

[ref36] Nagorsky E. Wiemeyer J. (2020). The structure of performance and training in esports. PLoS One 15:e0237584. doi: 10.1371/journal.pone.0237584, PMID: 32841263 PMC7447068

[ref37] Palomäki J. Laakasuo M. Salmela M. (2012). Losing more by losing it: poker experience, sensitivity to losses and tilting severity. J. Gambl. Stud. 30, 187–200. doi: 10.1007/s10899-012-9339-4, PMID: 23070722

[ref38] Palomäki J. Laakasuo M. Salmela M. (2013). 'This is just so unfair!': a qualitative analysis of loss-induced emotions and tilting in on-line poker. Int. Gambl. Stud. 13, 255–270. doi: 10.1080/14459795.2013.780631

[ref39] Parshakov P. Zavertiaeva M. (2018). Determinants of performance in eSports: a country-level analysis. Int. J. Sport Financ. 13, 34–51.

[ref40] Pedraza-Ramirez I. Musculus L. Raab M. Laborde S. (2020). Setting the scientific stage for esports psychology: a systematic review. Int. Rev. Sport Exerc. Psychol. 13, 319–352. doi: 10.1080/1750984X.2020.1723122

[ref41] Phan O. Prieur C. Bonnaire C. Obradovic I. (2019). Internet gaming disorder: exploring its impact on satisfaction in life in PELLEAS adolescent sample. Int. J. Environ. Res. Public Health 17:3. doi: 10.3390/ijerph17010003, PMID: 31861283 PMC6981998

[ref42] Pontes H. M. Griffiths M. D. (2014). Assessment of internet gaming disorder in clinical research: past and present perspectives. Clin. Res. Regul. Aff. 31, 35–48. doi: 10.3109/10601333.2014.962748

[ref43] Poulus D. Coulter T. J. Trotter M. G. Polman R. (2020). Stress and coping in esports and the influence of mental toughness. Front. Psychol. 11:628. doi: 10.3389/fpsyg.2020.00628, PMID: 32390900 PMC7191198

[ref44] Poulus D. Coulter T. Trotter M. Polman R. (2022a). Perceived stressors experienced by competitive esports athletes. Int. Journal. Esports 1.

[ref45] Poulus D. Coulter T. J. Trotter M. G. Polman R. (2022b). Longitudinal analysis of stressors, stress, coping and coping effectiveness in elite esports athletes. Psychol. Sport Exerc. 60:102093. doi: 10.1016/j.psychsport.2021.102093

[ref46] Reitman J. G. Anderson-Coto M. J. Wu M. Lee J. S. Steinkuehler C. (2020). Esports research: a literature review. Games Culture 15, 32–50. doi: 10.1177/1555412019840892

[ref47] Sharma M. K. Anand N. Amudhan S. Vashisht A. (2022). Online gaming and tilting: psychosocial exploration for promotion of emotional regulation. Int. J. Soc. Psychiatry 68, 699–701. doi: 10.1177/00207640211028602, PMID: 34187227

[ref48] Sharpe B. T. Besombes N. Welsh M. R. Birch P. D. (2022). Indexing esport performance. J. Elect. Gaming Esports 1, 1–13. doi: 10.1123/jege.2022-0017

[ref49] Sharpe B. T. Leis O. Moore L. Sharpe A. T. R. Seymour S. Obine E. A. C.. (2024b). Reappraisal and mindset interventions on pressurised esport performance. Appl. Psychol. 1–22. doi: 10.1111/apps.12544

[ref50] Sharpe B. T. Obine E. A. C. Birch P. D. J. Pocock C. Moore L. J. (2024a). Performance breakdown under pressure among esports competitors. Sport Exer. Performance Psychol. 13, 89–109. doi: 10.1037/spy0000337

[ref51] Smith M. J. Birch P. D. Bright D. (2019). Identifying stressors and coping strategies of elite Esports competitors. Int. J. Gaming Comp. Med. Simul. 11, 22–39. doi: 10.4018/IJGCMS.2019040102

[ref52] Stamatis A. Grandjean P. Morgan G. Padgett R. N. Cowden R. Koutakis P. (2020). Developing and training mental toughness in sport: a systematic review and meta-analysis of observational studies and pre-test and post-test experiments. BMJ Open Sport Exer. Med. 6:e000747. doi: 10.1136/bmjsem-2020-000747, PMID: 32577300 PMC7299040

[ref53] Tavakol M. Dennick R. (2011). Making sense of Cronbach’s alpha. Int. J. Med. Educ. 2, 53–55. doi: 10.5116/ijme.4dfb.8dfd, PMID: 28029643 PMC4205511

[ref54] Teng Z. Pontes H. M. Nie Q. Xiang G. Griffiths M. D. Guo C. (2020). Internet gaming disorder and psychosocial well-being: a longitudinal study of older-aged adolescents and emerging adults. Addict. Behav. 110:106530. doi: 10.1016/j.addbeh.2020.106530, PMID: 32683173

[ref55] Trotter M. G. Coulter T. J. Davis P. A. Poulus D. R. Polman R. (2021). Social support, self-regulation, and psychological skill use in e-athletes. Front. Psychol. 12:722030. doi: 10.3389/fpsyg.2021.722030, PMID: 34858261 PMC8632024

[ref56] Trotter M. G. Obine E. A. Sharpe B. T. (2023). Self-regulation, stress appraisal, and esport action performance. Front. Psychol. 14:1265778. doi: 10.3389/fpsyg.2023.1265778, PMID: 37885748 PMC10598391

[ref57] Türkay S. Formosa J. Adinolf S. Cuthbert R. Altizer R. (2020). See no evil, hear no evil, speak no evil: How collegiate players define, experience and cope with toxicity. Proceedings of the 2020 CHI conference on human factors in computing systems. 1–13).

[ref58] Viladrich C. Angulo-Brunet A. Doval E. (2017). A journey around alpha and omega to estimate internal consistency reliability. Anales De Psicologia 33:755. doi: 10.6018/analesps.33.3.268401

[ref59] Welsh M. R. Mosley E. Laborde S. Day M. C. Sharpe B. T. Burkill R. A.. (2023). The use of heart rate variability in Esports: a systematic review. Psychol. Sport Exer. 69:102495. doi: 10.1016/j.psychsport.2023.102495, PMID: 37665930

[ref60] Wood R. T. A. Griffiths M. D. Chappell D. Davies M. N. O. (2004). The structural characteristics of video games: a psycho-structural analysis. Cyberpsychol. Behav. 7, 1–10. doi: 10.1089/109493104322820057, PMID: 15006163

[ref61] Wu M. Lee J. S. Steinkuehler C. (2021). “Understanding tilt in esports: a study on young league of legends players” in Proceedings of the 2021 CHI conference on human factors in computing systems, 1–9.

