# Supplementary material for: Conceptualization and validation of the TILT questionnaire: relationship with IGD and life satisfaction
Source: Front Psychol. 2024 Jul 8;15:1409368. doi: 10.3389/fpsyg.2024.1409368 (PMC11260801; doi:10.3389/fpsyg.2024.1409368)
Supplement: Supplementary file 1 [file Table_1.pdf]

ANEX

TILTQ

Please indicate the extent to which you have experienced the following situations during a game in the last 15 days.

Legend (1=Completely disagree 2=Disagree 3=Neither disagree nor agree 4=Agree 5=Completely agree)

	1	2	3	4	5
I have lost because of things in the game I could not control.					
I have failed to make important moves-					
I have made mistakes in things I know I can do well.					
I have made wrong decisions.					
I failed even though I knew what I had to do.					
I have felt that I have more ability than I have been able to demonstrate.					
I have played frustrating games.					
I have felt that the game was not fair.					
I have exploded with rage.					
I have felt irritated.					
I have made decisions without thinking.					
I have made decisions without thinking.					
I have found it hard to concentrate.					
I have had mood swings due to the outcome of my games.					
I have felt that I have no energy.					
I have felt that I have been on a losing streak that I could not get out of.					
I have played hastily.					
I have continued to play even though I did not feel like it.					
I have written off games as lost.					
